# Patient-specific Computational Hemodynamic Analysis for Interrupted Aortic Arch in an Adult: Implications for Aortic Dissection Initiation

**DOI:** 10.1038/s41598-019-45097-z

**Published:** 2019-06-13

**Authors:** Liqing Peng, Yue Qiu, Zhigang Yang, Ding Yuan, Chenzhong Dai, Da Li, Yi Jiang, Tinghui Zheng

**Affiliations:** 1 Department of Radiology, West China Hospital, Sichuan University, Chengdu, 610041 China; 2 Department of Applied Mechanics, Sichuan University, Chengdu, 610065 China; 3 Department of Vascular Surgery, West China Hospital, Sichuan University, Chengdu, 610041 China

**Keywords:** Aortic diseases, Computational science

## Abstract

The guideline for the treatment of interrupted aortic arch (IAA) in adults has not been established although most centers tend to propose surgery. There is no clear evidence for the preferred selection of surgical repair versus conservatively medical treatment for the uncertain effects of both treatments. However, reports of sporadic aortic dissection (AD) of descending aorta (DAo) in IAA in adults before surgery drew our attention. It is quite perplexing because there seems to be no risk factors for the development of AD at DAo such as long-term uncontrolled hypertension, atherosclerosis, aortic aneurysm or genetic disorder. In this paper, we carried out the numerical investigation on the hemodynamics in a patient-specific IAA model, which was reconstructed from computed tomography images. Hemodynamic parameters including the flow pattern, pressure distribution, and wall shear stress (WSS) indicators were obtained. The simulation revealed that the jet flows from the collateral arteries (CAs) induced risk hemodynamic forces on the lumen wall including high time-averaged wall shear stress (TAWSS), high pressure and rapid change of WSS direction throughout the cardiac cycle. Moreover, it is found that only a jet flow which circumferentially washes out the aortic wall might cause tears on the wall. It is concluded that the specific geometrical features of the extensive major CAs might result in the risky hemodynamics leading to the initiation and development of AD in this particular IAA patient. CFD analysis in IAA can provide a clinical reference, and the results should be further studied in depth in the future.

## Introduction

Interrupted aortic arch (IAA) is defined as a complete loss of luminal and anatomic continuity between ascending and descending parts of the aorta^1^. IAA is an uncommon congenital malformation of the aortic arch that occurs in 3 per million live births and accounts for only 1% of all congenital heart disease^1^. IAA is much more frequently encountered in the neonate, and immediate surgical intervention remains the only therapy. The prognosis of patients with IAA is very poor as congestive heart failure develops in neonates who are left uncorrected, leading to 90% mortality within 4 days of birth^2^. As for IAA in adult patient, it is extremely uncommon with only 40 cases documented so far. Of the 40 reported adult patients with IAA, only 3 presented with congestive heart failure as IAA in the adult usually develops extensive collateral arteries^1–4^. IAA in adult patients can live for many years asymptomatically due to existence of extensive collateral arteries (CAs), and whether these patients should be treated medically or with surgical repair has not been determined. Of the 40 patients reported, more than half were treated surgically^5,6^. Öztürk *et al*.^5^ reported medical treatment of an adult with uncorrected isolated IAA resulted in no complications after 4 years of follow-up. Follow up of 8 cases of IAA in adults shows higher incidence of surgical sequelae in older patients, and the widely varied results of both surgical and conservative treatment. There is still no evidence regarding how to select patients who should receive medical treatment and who are proper candidates for surgery in clinical practice^5^.

In adults, the clinical presentation of IAA varies from the absence of symptoms to a headache, malaise, hypertension, claudication, differential blood pressure between the upper and lower extremities, limb swelling, congestive heart failure and aortic dissection. As the most severe and high-risk presentation and complication, aortic dissection in adult in IAA requires special attention and concern. According to literature, 5 cases of IAA in adult patients were reported with type B aortic dissection^6–10^. It is very interesting that the dissection initial intimal tear occurred at upper segment of descending thoracic aorta in all the 5 patients because the blood pressure in descending aorta is hypotension other than hypertension. There seems to be no risk factors for descending aortic dissection such as long-term uncontrolled hypertension, atherosclerosis, aortic aneurysm or genetic disorder that affects the body’s connective tissue like Marfan syndrome^6,7^. Finding out the risk factors leading to dissection of descending aorta in these patients is very interesting and clinically significant. According to literature, computational fluid dynamics investigations have been widely used to clarify the characteristics of cardiovascular flow to explain the development and progression of different diseases^11,12^. We hypothesize that there might be abnormal hemodynamics which predisposes to the descending aortic dissection. Thus, we conducted a computational fluid dynamics study in IAA of an adult to seek to determine the hemodynamic risk factors for aortic dissection based on computed tomography angiography (CTA) data sets.

## Results

### Geometry features

**Figure 1:**
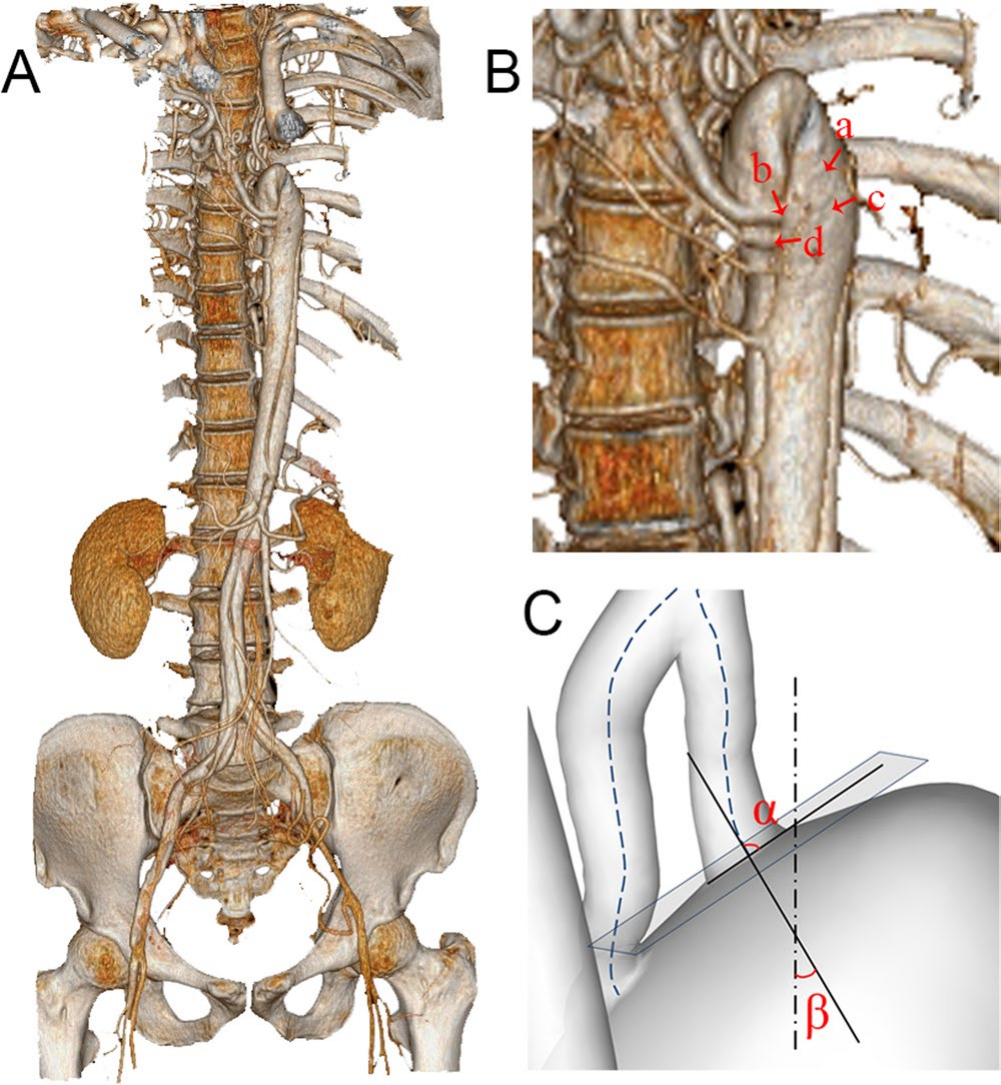
3D reconstruction of IAA model. (**A**) The reconstructed geometry with dissection using CT images; (**B**) Four tears a, b, c and d are marked with red arrows; (**C**) Angles α (the angle between the axis line and the tangent plane) and β (the angle between the axis line and the vertical axis) of CA_1_ are demonstrated. (CT = computed tomography; CA = collateral artery).

**Figure 2:**
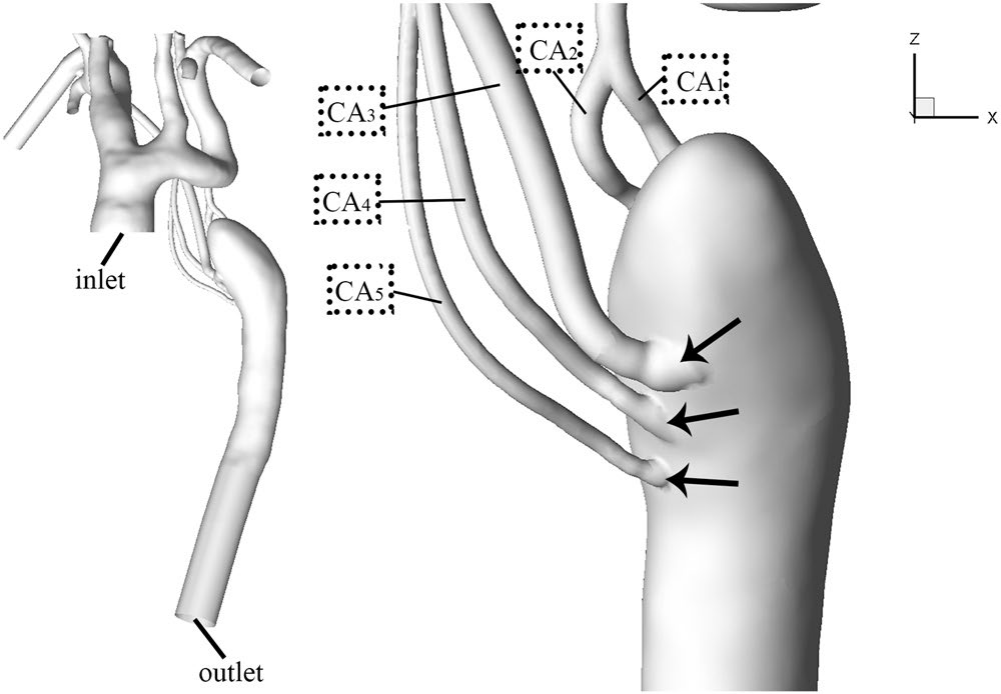
3D reconstruction of IAA model. The reconstructed model for CFD calculation and number of five collateral arteries based on CT images. The black arrows represent vessel dilation (CFD = computational fluid dynamics; CT = computed tomography).

As shown in the reconstructed three-dimensional geometrical model of the IAA patient (Fig. 1A), all the collateral arteries (CAs) experienced more or less vessel dilation at the anastomoses where the CAs enter the DAo (as indicated by the arrows in Fig. 2). It is apparent that the entire descending aorta bent backward, and the dissection longitudinally expanded to the iliac arteries and laterally terminated at each CA. In addition, the false lumen (FL) originated from the anterior left lateral wall of the DAo near CA3, CA4, and CA5, then the FL propagated both proximally to the crescent top of the aorta and distally but slowly turn to the right side of the aorta (Fig. 1A). The FL was smaller than the true lumen (TL), with a maximum hydraulic diameter of 7.32 mm, compared with 18.61 mm for the TL. The entire segment of the FL starting from the crescent top of the aorta to the iliac arteries was 44.8 cm in length. There were four tears confirmed from the CTA images whose sizes were 2.86 mm, 3.34 mm, 1.72 mm and 1.59 mm, respectively. The tears were named as tear A, B, C, D in sequence and then projected to the aortic wall as shown in Fig. 1B, among which tear A located at the top segment of the aorta, tear B, the largest one, at the dilated end of CA3, tear C at the aorta wall which was only 3.2 cm away from the crescent top of the DAo and Tear D, the smallest one, at the dilated end of CA4, respectively.

Table 1 listed more specific geometrical characteristics of each CA. The average diameters of the CAs were 3.40 mm, 4.61mm, 5.72 mm, 3.52 mm and 2.76 mm, respectively. The angle α (the axis line of each CA with respect to the tangent plane of DAo were 58.57°, 37.54°, 29.67°, 34.29° and 7 52.03°, respectively, and the angle β (the axis line of each CA with respect to the vertical axis of DAo) were 39.80°, 59.58°, 85.50°, 78.18° and 86.93°, respectively (Table 1) (Fig. 1C).

**Table 1:** The geometry of five main collateral arteries.

Collateral arteries	Diameter (mm)	α (°)	β (°)
CA_1_	3.40	58.57	39.80
CA_2_	4.61	37.54	59.58
CA_3_	5.72	29.67	85.50
CA_4_	3.52	34.29	78.18
CA_5_	2.76	52.03	86.93

Note: Angle α indicates the angle between the axis line of each collateral artery and the tangent plane, while Angle β indicates the angles between the axis line of each collateral artery and the center line of descending aorta. CA = collateral arteries.

### Flow pattern

**Figure 3:**
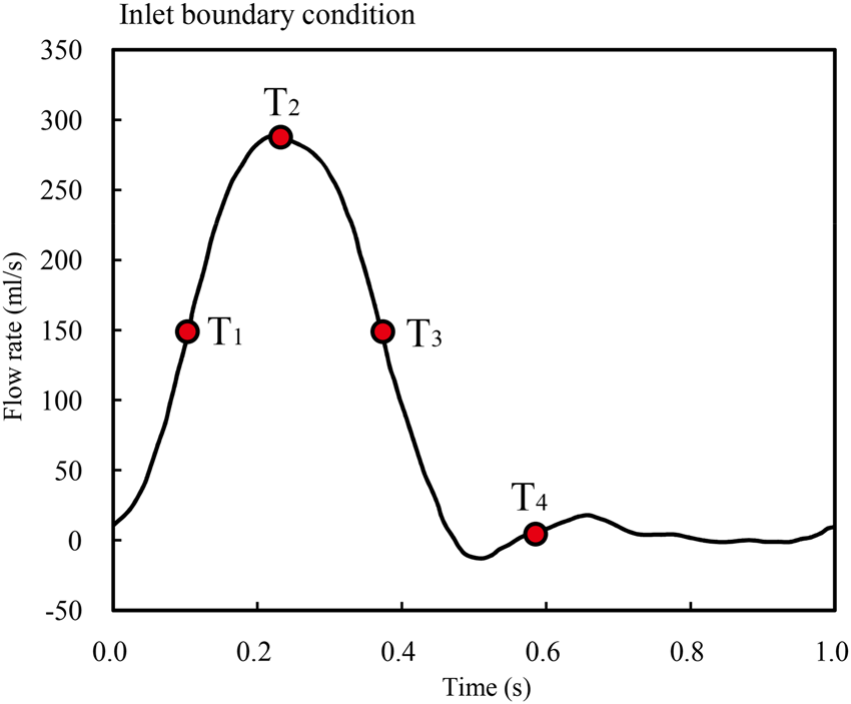
Inlet boundary condition. Four specific time points marked with red circle are chosen for analysis: T1 = 0.11 s; T2 = 0.22 s; T3 = 0.36 s; T4 = 0.58 s.

Four-time points during cardiac cycle were selected for analysis. T1, early-systole; T2, mid-systole; T3, late-systole, and T4, early diastole (Fig. 3).

At early-systole, no reverse flow from the aorta to any CA was observed, blood flow from CA_3_ split into two parts, with one part spirally going up to the DAo upper end, the remaining flow down to distal DAo. The strong spiral flow only appeared in the upper 2 cm segment of the DAo and the flow velocities in the whole DAo were small. At mid-systole, the flow velocities in all CAs increased, which resulted in flow from CA_5_ also first washed against the lumen wall and then went downstream. The area of strong spiral flow extended to about upper 6 cm segment of the DAo. At late-systole, the strong spiral flow region further extended to upper 8 cm in the length, the flow near the tears became quite complicated and chaotic. In addition, the flow velocities in CA_5_ decreased sharply and became almost close to zero. At early diastole, the strong spiral flows were present in the whole DAo segment, and reverse blood flow to all CAs except CA_3_ were observed. Namely, only blood from CA_3_ remained flowing outward to the DAo throughout the cardiac cycle.

**Figure 4:**
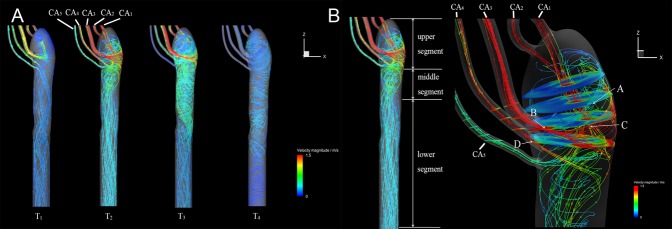
Streamlines in the descending aorta and the velocity magnitude are differentiated with the color bar. (**A**) Streamlines at the four time points; (**B**) Detailed view of the tears at T2. The white arrows point to the tear locations.

Figure 4B shows the close-up streamlines near the tears at mid-systole. The blood from all CAs formed jet injection, each jet then became its own main flow channel. To be notified that only blood from CA_5_ and CA_1_ directly flow into the lumen with no stick to the vessel wall, the jet flow from other CAs all hit the aorta wall but in different adhesion mode. First, the blood from CA_3_ and CA_4_ both laterally sprayed to the aorta wall. Partial blood from CA_4_ went up circularly and spirally while the remaining joined the blood from other CAs spirally flow downstream, and finally stabilized to be axial and laminar flow. But because the jet flow from CA_4_ was not strong enough to go up, after a transient adherence to the vessel wall the blood spirally flow downstream. By contrast, due to its small angle between CA_2_ with the lumen axis, after it sprayed to the wall, it directly flowed downstream along the wall. To be notified that, tears A, B, and C all located at the impact area of the jet flow from CA_3_, and tear B and C located at the high-speed flow channel while tear A at the low-speed upward flow. In addition, tear D located in the main channel flow from CA_4._

### Pressure distribution

**Figure 5:**
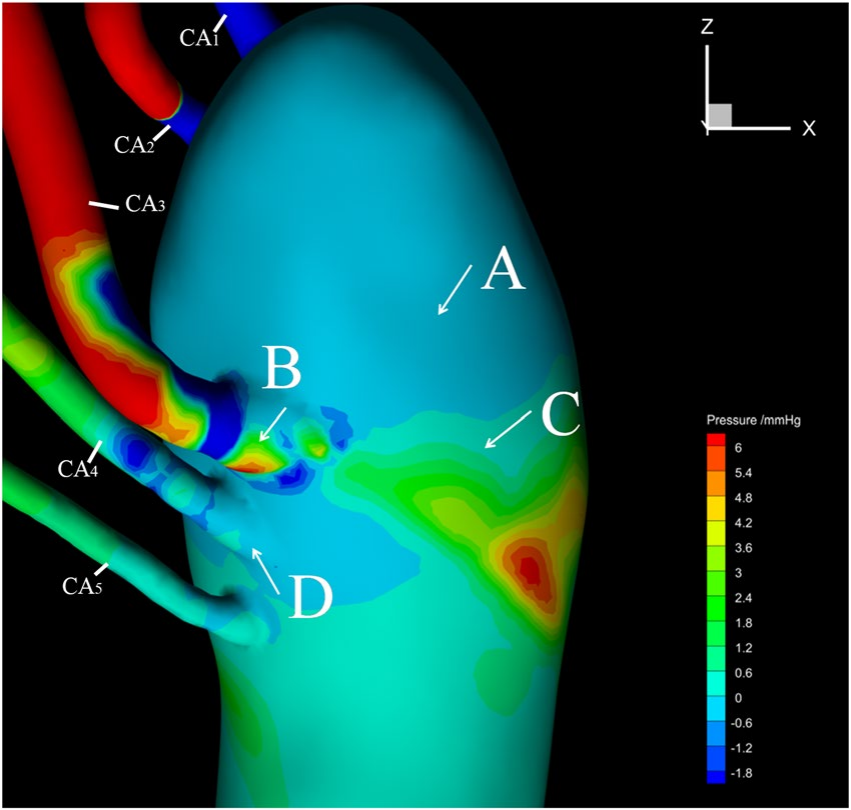
Pressure distribution of the tears at mid-systole (T2). The white arrows point to the tear locations.

Figure 5 shows the pressure distribution at the mid-systole. The pressure at the outlets of CA_1_, CA_2_ experience a sharp decrease from positive 6 mmHg to negative 1.8 mmHg and then increase to negative 0.6 mmHg in the top section of the aorta which indicates a flow reverse there. The outlet area of CA_3_ has a very complicated and non-uniform pressure distribution where pressure first decreased to a negative value, then increased to positive values, but decreased to negative and finally increased to positive again which resulted in large pressure gradients. Although on the whole, the pressure at CA_4_ and CA_5_ were lower than other CAs, adverse pressure gradient also appeared at the anastomoses between CA_4_ and the aorta. Tear A and D located in the region of negative pressure while the pressures near tear B and C were positive and the pressure gradient was large there.

### WSS-based indicators

**Figure 6:**
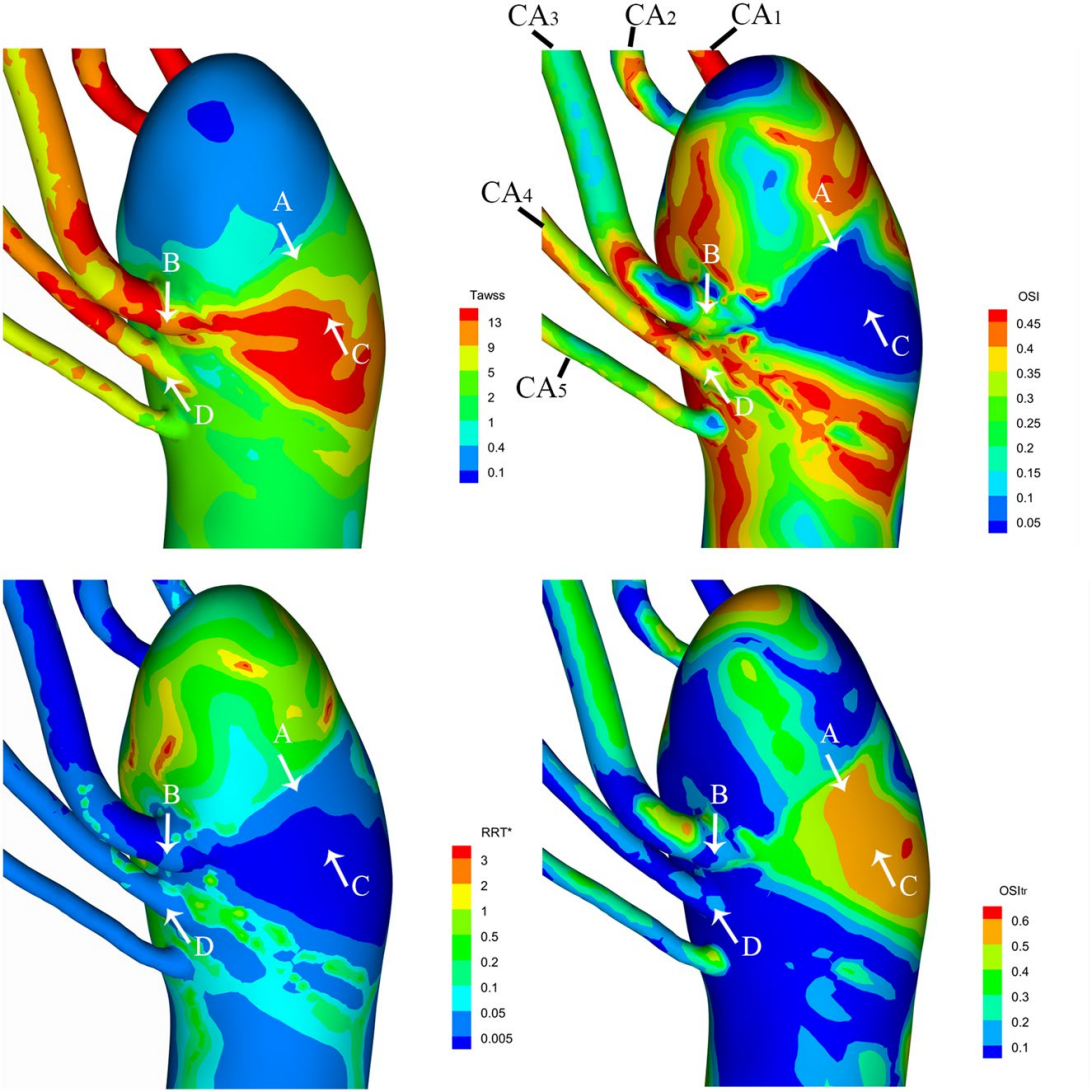
Contour map of the WSS-based indicators of the tears. The white arrows point to the tear location.

Based on the changes of WSS throughout an entire cardiac cycle, some important hemodynamic indices including TAWSS, OSI, RRT, and OSItr are obtained. Figure 6 shows the distributions of these indices and zoom-in views near collateral artery outlets are also present.

Table 2 shows the values of the hemodynamic parameters at the projection zones of all tears.

**Table 2:** Values of the hemodynamic parameters at the projection zones of all tears.

Position	TAWSS (Pa)	OSI	RRT*	OSItr
A	2.69	0.03	0.011	0.56
B	9.25	0.34	0.009	0.15
C	14.27	0.01	0.002	0.57
D	7.76	0.34	0.011	0.08

The TAWSS (time-averaged WSS) is used to evaluate the average WSS on the vessel wall throughout the cardiac cycle. The definition is as follows:

$${\rm{TAWSS}}=\frac{1}{{\rm{T}}}{\int }_{0}^{{\rm{T}}}|{\rm{WSS}}({\rm{s}},{\rm{t}})|\cdot {\rm{dt}}$$

${\rm{T}}$ is the heartbeat period.

We define areas of the TAWSS values below 0.4 Pa as low WSS zones while above 5 Pa as high WSS zones^13^. It can be seen the low shear zone only appears in the recirculation region of the upper segment of the descending aorta and in a small area at descending aorta downstream. All the anastomoses between the descending aorta and each collateral artery were covered with high WSS where the peak WSSs in the intersection segments between the collaterals CA_1_, CA_2_, CA_3_ and the aorta reach as high as 43.4 Pa, 53.5 Pa, 27.1 Pa respectively. The jet flow from collaterals A2 and A3 increase the WSSs in their impact zones on the aorta wall as high as 23.8 Pa and 17.9 Pa, respectively. The close-up views of the TAWSS distribution near each tear indicate that the WSS at tear A is 2.69 Pa which is in the normal range of TAWSS, the WSSs at tear B, C, D are 9.25 Pa, 14.27 Pa, and 7.76 Pa, respectively.

The OSI (oscillatory shear index) is a constantly used index to evaluate the axial directional change of WSS during a cardiac cycle and is defined as:

$${\rm{OSI}}=0.5\,[1-(\frac{|1/{\rm{T}}{\int }_{0}^{{\rm{T}}}{\rm{WSS}}({\rm{s}},{\rm{t}})\cdot {\rm{dt}}|}{1/{\rm{T}}{\int }_{0}^{{\rm{T}}}|{\rm{WSS}}({\rm{s}},{\rm{t}})|\cdot {\rm{dt}}})]$$

OSI ranges from 0 to 0.5, a zero value of OSI corresponds to unidirectional shear flow and when a purely oscillatory shear case happened OSI value becomes to 0.5, the OSI at the top 6 cm segment of the aorta are non-uniformly distributed and have large values due to the complicated and unstable flow there. But when flow becomes stable and maintains axial flow at aorta downstream the OSI values maintain near zeros. As is shown in the contour maps of streamline (Fig. 4), reverse flow happens over the whole cardiac cycle, so OSI values in some region reach as high as 0.45. But it is no surprise that the OSIs in the region where jet flows from collateral A3 flush the aorta is almost zero. More specifically, the zoom-in view of the OSI contour map near the tears shows that the OSI at tears A and C are close to zero, but OSIs at tear B and D reach as high as 0.34.

The RRT (relative residence time) is an index that can indicate the residence time of particles near the vessel wall with a definition as:

$${\rm{RRT}} \sim \frac{1}{(1-2\cdot {\rm{OSI}})\cdot {\rm{TAWSS}}}$$

As can be seen from the contour map, the RRT values at all tears are very low while not to our surprise that the RRT values at the top section where the flow is re-circulating and stagnant are high.

TransWSS is calculated as the time-average of WSS components perpendicular to the mean flow direction, it is introduced as a better metric to investigate the multi-directionality of flow fields than OSI^14^. The TransWSS value can take from zero to TAWSS, so we normalized the TransWSS with its corresponding TAWSS. And this parameter is OSItr which ranges from 0 to 1^15^.

$${\rm{TransWSS}}=\frac{1}{{\rm{T}}}{\int }_{0}^{{\rm{T}}}|{\rm{WSS}}({\rm{s}},{\rm{t}})\cdot ({\rm{n}}\times \frac{\frac{1}{{\rm{T}}}{\int }_{0}^{{\rm{T}}}{\rm{WSS}}({\rm{s}},{\rm{t}})\cdot {\rm{dt}}}{|\frac{1}{{\rm{T}}}{\int }_{0}^{{\rm{T}}}{\rm{WSS}}({\rm{s}},{\rm{t}})\cdot {\rm{dt}}|})|$$

${\rm{OSItr}}=\frac{{\rm{TransWSS}}}{{\rm{TAWSS}}}\,$Peak OSItr appears at the flow channel of collateral CA_3_ because of small changes in the direction of high-speed near-wall flow. For the same reason, the OSItr at tear C reaches as high as 0.56. Recall that tear A locates at the recirculation region, accordingly although the change of flow direction is small, it lasts over a larger portion of the cardiac cycle, so the OSItr at tear C also reach 0.67. tears B and D both locate at the end of the collateral artery and the OSItr at are 0.15 and 0.08, respectively.

## Discussion

Abnormal hemodynamic forces, as well as disturbed flow, are suggested to play a key role in the initiation and development of AD^16,17^. For example, a long-time exposure to abnormal WSS retards the endothelial cell functions, such as proliferation, apoptosis, migration, permeability, and remodeling^18,19^. High spatial and temporal changes of WSS accompanied with disturbance flow damages the physiological processes such as wall remodeling, inflammation, and mass transport from the blood to the vessel wall^20^. In addition, chronic exposure to high pressure leads to wall thickening, fibrosis, calcification, and extracellular fatty acid deposition^21^.

The current study revealed that there were three features worth noting for the flow field of this IAA patient. First, the ascending aorta supplies blood to the DAo through small diameter collaterals which result in high-speed flow in the CAs. Second, some collaterals are seriously eccentric causing its high-speed flow directly injects into the aorta. Third, the upper segment of the descending aorta suffered from highly disturbed flow including helical, vortical and stagnant flow during the cardiac cycle. As a result, the top 2 cm segment of the DA suffered from abnormal hemodynamic stresses on the vessel wall including high pressure and WSS over the entire cardiac cycle. For example, physiological WSS values typically range from 0.4 to 2.0 Pa in aorta^22^, but the TAWSS at all tears are beyond the upper limit. Especially the value at Tear C reached as high as 14.27 Pa. In addition, the disturbed flow there also leads to high OSI or OSItr values near the tears which indicates that flow is not purely axial forward, they are either pulsatile with reversal (tear B and D with high OSI) or multi-directional disturbed (tear A and C with high OSItr). In a word, the hemodynamics within the DAo for this IAA patient is risky for the initiation and development of AD.

Geometric configurations are decisive to the nature of hemodynamic patterns. Therefore, it is likely that morphological factors are important determinants in the initiation of AD for this IAA patient. AD occurs when an injury to the innermost layer of aorta cause a tear which allow blood leaks through, and then the blood flow between the layers of the aortic wall finally force the layers apart, the dissection propagates proximally and distally. As a matter of fact, there are four tears observed in the DAo of this IAA patient, among which three tears (Tear B, C, and A) located at the wash region of CA_3_ jet flow and the rest one located at the impact region of CA_4_ jet flow. By comparing the three CAs (CA_3_, CA_4_ and CA_5_) with the others (CA_1_, CA_2_), it is not hard to find out that due to their large angle with the DAo axis, both their jet flow circumferentially flushed the wall, while the jet flow from others either directly flow into the lumen (CA_1_ and CA_2_). In another word, whether it may cause a tear or not depends on how the jet flow washes the vessel wall.

In conclusion, the specific geometrical features of the extensive major CAs caused the risky hemodynamics which resulted in the injury to the intima of this patient-specific. More specifically, two factors make IAA in the adult patient more susceptible to AD. First, whether the jet flow from a CA impinges on the lumen wall or not which will induce high WSS and pressure there. Second but most important is how the jet flow wash the wall, circumferentially or axially? If it is in a circumferential way, it might have a greater chance to develop AD in the future. CFD analysis in IAA can provide a clinical reference for risk evaluation, and the results should be further studied in depth in the future.

## Methods

### Geometry model

This study was conducted in accordance with the principles of the Declaration of Helsinki and met the requirement of medical ethics. The Ethical Review Committee of the West China Hospital of Sichuan University (Chengdu, Sichuan, China) approved this research. As our study was purely observational and retrospective in nature and used anonymized data, patient approval and informed consent were waived. Patient-specific data sets were provided by the West China Hospital of Sichuan University (Chengdu, Sichuan, China) and included computed tomography angiography (CTA) data.

Thin-slice CTA data was obtained with a multi-slice computed tomography scanner (Somatom Definition Flash, Siemens Medical Solutions, Germany) CTA images of the IAA were obtained to reconstruct the three-dimensional IAA geometry using the commercially available software Mimics (version 14.0; Materialise, Plymouth, Mich). Because the entire geometry is complex and only the hemodynamic environment at the segment where aortic dissection begins is our concern, some reasonable simplifications are adopted. (1) It is assumed that the aorta geometry experiences subtle change before and after the occurrence of dissection and could be ignored according to the clinical experience of radiologists specializing in cardiovascular imaging. Accordingly, we build the model excluding the dissection flap. (2) Only five main collateral arteries (CAs) feeding upper part of descending thoracic aorta were preserved and the small ones were ignored. We named the five CA as CA_1_, CA_2_, CA_3_, CA_4_, and CA_5_, respectively (Fig. 2).

### Governing equation

The blood flow was assumed isotropic, homogeneous, incompressible and Newtonian and the corresponding governing equations are given as follows:

1 $$\rho \frac{\partial \overrightarrow{u}}{\partial t}+\rho (\overrightarrow{u}\cdot {\nabla })\overrightarrow{u}+{\nabla }p-\mu \triangle \overrightarrow{u}=0\,$$

2 $${\nabla }\cdot \overrightarrow{u}=0\,$$

where $\overrightarrow{u}$ and *p* represent, respectively, the fluid velocity vector and the pressure. *ρ* and *μ* are the density and dynamic viscosity of blood, which were given to 1050 *kg*/*m*^3^ and 3.5 × 10^−3^ *kg*/*m s*, respectively.

### Boundary condition

The inflow waveform which is shown in Fig. 2 was adapted from Olufsen *et al*.^23^. So, based on the inlet aortic diameter (32 mm), the corresponding mean Re number is 1090. Every outlet was defined as outflow and the flow ratio was set as 6.48% in bilateral carotid arteries, 18.15% in bilateral subclavian arteries, 8.73% in bilateral mammary arteries and 33.28% in descending aorta (DAo)^24^.

### Numerical simulation

The flow visualization and analysis were completed by the computational fluid dynamics software Ansys FLUENT 12.0 based on the finite volume method. A default implicit 3D solver was applied. Discretization of the equations involved a second order upwind differencing scheme, SIMPLEC was adopted for the pressure-velocity correction and the residual error convergence threshold was set to 10e-5.
